# Supplementary material for: The Construction and Exploration of a Comprehensive MicroRNA Centered Regulatory Network in Foxtail Millet (Setaria italica L.)
Source: Front Plant Sci. 2022 May 6;13:848474. doi: 10.3389/fpls.2022.848474 (PMC9121102; doi:10.3389/fpls.2022.848474)
Supplement: Supplementary file 4 [file Data_Sheet_4.zip › Sit-miR2275d_Seita.4G084400.1_215_TPlot.pdf]

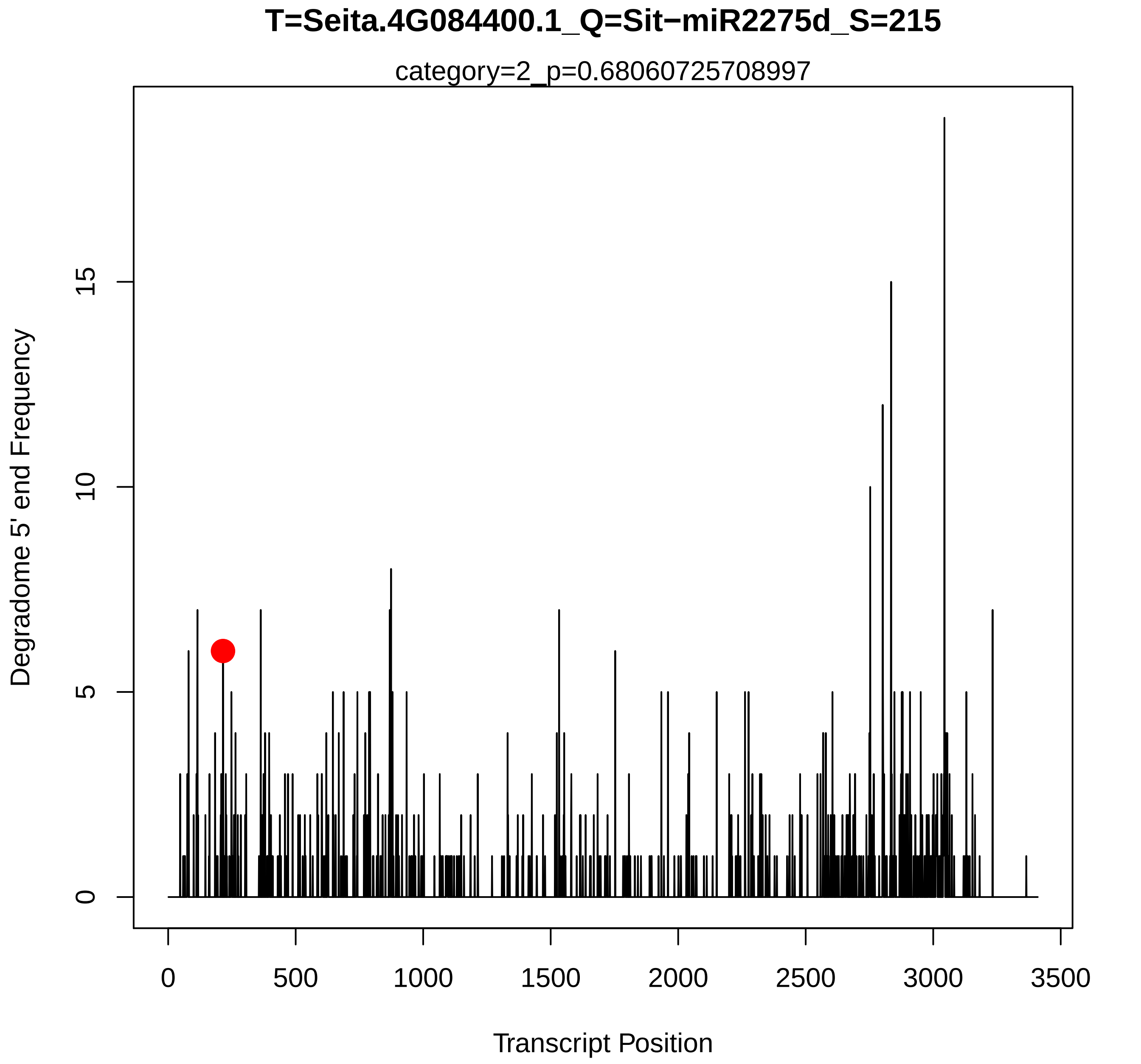

T=Seita.4G084400.1_Q=Sit−miR2275d_S=215
category=2_p=0.68060725708997
Degradome 5' end Frequency
0
5
10
15
0
500
1000
1500
2000
2500
3000
3500
Transcript Position
